# Volumetric Analysis of Ridge Preservation Using Bio-Oss^®^ Collagen: A Retrospective Cohort Study Based on CBCT and Panoramic Radiographs

**DOI:** 10.3390/medicina62050888

**Published:** 2026-05-05

**Authors:** Katharina Hartmann, Markus Tröltzsch, Amely Hartmann, Sven Otto, Matthias Tröltzsch

**Affiliations:** 1 Department of Oral and Maxillofacial Surgery and Facial Plastic Surgery, LMU Medizin, LMU University Hospital, Ludwig-Maximilians-Universität München, Lindwurmstraße 2a, 80337 Munich, Germany; 2 Centre for Oral, Maxillofacial and Facial Reconstructive Surgery, Maximilianstraße 5, 91522 Ansbach, Germany; 3 Clinic for Oral Surgery and Implantology Dr. Seiler und Kollegen MVZ, 70794 Filderstadt, Germany; 4 Department of Oral and Maxillofacial Surgery, Plastic Surgery, University Medical Centre of the Johannes Gutenberg University of Mainz, 55131 Mainz, Germany

**Keywords:** alveolar ridge resorption, xenogeneic bone substitute, volumetric analysis, cone-beam imaging, xenogeneic collagenous graft, tooth extraction

## Abstract

*Background and Objectives:* Tooth loss affects quality of life and chewing ability and is associated with natural ridge resorption after extraction. Implants are a viable option for the anchorage of removable or fixed prostheses. Successful implant placement requires adequate bone availability. To minimize bone loss after extraction and to avoid the need for additional augmentation before implant placement, ridge preservation techniques are employed. The aim of this study was to assess volume changes in extraction sockets after ridge preservation with a collagen-based bovine/porcine xenogenic material, Bio-Oss^®^ Collagen (Geistlich, Switzerland), in molar and premolar regions. *Materials and Methods:* A retrospective study was designed and implemented. Subjects who underwent tooth extraction and consecutive ridge augmentation with Bio-Oss^®^ Collagen between 2018 and 2022 and complied with the inclusion criteria were selected. The volume of the tooth root prior to extraction (alveolar socket volume surrogate) was estimated from pre-extraction CBCT scans and panoramic radiographs (predictor variable). The volume of the socket after extraction and ridge preservation was measured in CBCT datasets (outcome variable). The results were tabulated and analyzed (*p* < 0.05). *Results:* The study sample was composed of 80 subjects (37 female, 43 male; 20 premolars, 60 molars; average age: 59 ± 12.5 years). Of those, 60 cases qualified for comparative analyses (27 female, 33 male; 15 premolars, 45 molars; avg. age 59 ± 12.7 years). Compared with the pre-extraction alveolar socket volume in this subset of 60 subjects (maxillary premolar: 195.20 ± 33.40 mm^3^, maxillary molar: 470.41 ± 99.92 mm^3^, mandibular premolar: 220.42 ± 102.03 mm^3^, mandibular molar: 544.76 ± 137.32 mm^3^), ridge preservation cases still exhibited a volume loss of approximately 3–18% due to residual resorption depending on the location of the augmentation site (volume after ridge preservation: maxillary premolar: 192.07 ± 63.50 mm^3^, maxillary molar: 381.96 ± 81.38 mm^3^, mandibular premolar: 199.86 ± 73.70 mm^3^, mandibular molar: 475.85 ± 152.26 mm^3^. The highest resorption rates were observed in maxillary molar sites (approximately 18%), whereas maxillary premolar sites showed the lowest rates (around 3%). *Conclusion:* The study demonstrates that ridge preservation with the xenograft Bio-Oss^®^ Collagen (Geistlich, Switzerland) can reduce ridge resorption following tooth extraction.

## 1. Introduction

Tooth loss can negatively affect the quality of life and chewing ability [1]. Implants can restore both the function and aesthetics of the stomatognathic system, offering a range of options and possibilities [2]. The success of oral implants relies on the quality and quantity of the residual alveolar bone [3].

Naturally, tooth loss leads to progressive ridge resorption as part of the alveolar remodeling process [4,5,6,7,8,9,10,11]. This process is dynamic and typically lasts approximately 3 months [4,5,12,13]. It leads to significant dimensional alterations, primarily observed as a reduction in bone volume. Resorption is more pronounced horizontally than vertically. In the bucco-oral dimension, the ridge typically decreases by around 4 mm, corresponding to a 30–60% reduction, whereas vertical bone loss averages 1–1.5 mm, or roughly 10–20% [10,11,14].

The buccal alveolar plate is particularly susceptible to resorption, as it depends on the blood supply from the periodontal ligament and the periosteum, which are disrupted by the extraction [4,5,6,10,15]. It is critical to maintain the alveolar bone support of the buccal plate after extraction for superior implant site development [16,17].

Therefore, implementing measures to prevent or reduce post-extraction bone loss should be considered essential after tooth extraction. One such measure is ridge preservation, which involves techniques aimed at minimizing alveolar ridge resorption. The main goal is to maintain a ridge with a healthy bone structure and sufficient volume, enabling successful implant placement and osseointegration without the need for further augmentation procedures [18].

During ridge preservation, the extraction socket is filled with an appropriate grafting material, which stabilizes the initial blood clot and supports the vascularization of the surrounding bone [18,19,20]. Socket augmentation can be performed either with a graft material alone or in combination with membranes, utilizing either primary wound closure or more complex soft tissue management. Under optimal conditions, the biomaterial used for ridge preservation becomes incorporated into the alveolar architecture without impairing it, directing bone regeneration toward re-establishing the socket’s original volume. Evidence indicates that the structural integrity of the alveolar ridge is compromised in the absence of post-extraction bone grafting [10,14].

Several types of graft materials are available, including autogenous, xenogeneic, and synthetic grafts [21,22,23,24,25,26,27]. Collagenated xenografts are a type of grafting material that exhibit promising results in ridge preservation [28,29,30]. Within this group, numerous products from different manufacturers and different xenogenic origins are available. Each material exhibits distinct biological behavior and integration kinetics. Most of the available evidence is derived from animal studies or small human studies with cohorts of fewer than 30 patients [31,32,33]. Therefore, it is impossible to generalize published results from one material to another.

There is still a lack of knowledge regarding the achievable bone quality and volume [34]. Volume analyses are rarely performed because they usually require a cone-beam CT (CBCT), which is not always clinically indicated and may expose patients to unnecessary radiation. For ethical reasons, mostly only simple radiographs, such as periapical or panoramic radiographs, are obtained in clinical and scientific sessions.

In several studies, only width and length measurements were performed to evaluate the efficacy of ridge preservation [19,26,31,35]. However, volume analyses are the only effective method to confirm the true efficacy of alveolar grafting, revealing both horizontal and vertical volume gain or loss. The use of the volumetric approach to analyze the effectiveness of ridge preservation procedures is both technically challenging and bears inherent methodological inaccuracies. Software-based volumetric analyses of CBCT data have evolved from mainly manually driven to AI (artificial intelligence) controlled methods [36,37,38,39]. The manually controlled approach has been pre-validated [36,37,38,39]. Present and future studies may resort to AI-driven analyses, the reliability of which remains to be proven. The demand for volumetric analysis on the one hand and the availability of 2D radiography on the other hand require alternative analytic concepts [37].

The aim of this retrospective cohort study was to quantitatively assess volumetric changes in the alveolar ridge following socket preservation with a collagenated bovine xenograft (Bio-Oss^®^ Collagen, Geistlich, Switzerland; BO) using various radiographic imaging modalities (cone-beam computed tomography (CBCT) or panoramic radiographs (PR)) through manually controlled, validated volume measurements. It was hypothesized that the alveolar volume, defined as the pre-extraction root volume, could be maintained by alveolar grafting using a collagenated bovine xenograft. A secondary hypothesis was that patient- and/or treatment-related factors could be associated with the degree of volumetric preservation.

## 2. Materials and Methods

To address the research question, a retrospective cohort study was designed and implemented. All patients who visited a private practice for oral and maxillofacial surgery in Ansbach, Germany, between 2018 and 2022 and met the inclusion criteria were included in the study. The inclusion criteria were: (1) age > 18 years, (2) carious or periodontally compromised premolars or molars, (3) ridge preservation performed with Bio-Oss^®^ Collagen (Geistlich, Wolhusen, Switzerland, BO), and (4) no other form of augmentation in the region of interest. The exclusion criteria were: (1) severe periodontitis, (2) current anti-resorptive medication, (3) absence of preoperative radiographic data and/or incomplete clinical documentation, and (4) objection to study inclusion and data analysis (even if anonymized). The study was conducted in accordance with ethical guidelines, and institutional review board approval was obtained (Ethics Committee of the Ludwig-Maximilians University of Munich, application number 23-0322).

### 2.1. Variables

The predictor variable was the original volume of the tooth roots within the alveolar ridge (apex to crestal bone) in mm^3^ before extraction, serving as a surrogate for the ideal volume.

The primary outcome variable was the postoperative alveolar volume (mm^3^) after ridge preservation. Importantly, the postoperative measurement included the periapical region, which is frequently affected by pre-existing periapical osteolysis before extraction, and the periodontal ligament space. Because these osteolytic areas and the periodontal ligament space were subsequently filled with newly formed bone and/or Bio-Oss Collagen, it was expected that the postoperative alveolar volume might exceed the original root volume in some cases.

Secondary outcome variables included age (interval), exact tooth location (ordinal), the reason for tooth extraction (ordinal), diabetes (binary), anticoagulant therapy (binary), and the success of consecutive implant placement after healing. Covariates were sex, perioperative antibiotic administration (binary), the presence of periodontitis (binary), smoking status (binary) and the use of anticoagulants.

### 2.2. Treatment Protocol

Preoperative X-rays (CBCT or PR—conventional or digital) and informed consent were obtained prior to all surgeries. After local disinfection of the oral cavity with chlorhexidine mouth rinse, local anesthesia was applied as needed using articaine with epinephrine (Ultracain DS forte 1:100,000/1:200,000). The periodontal marginal ligament was incised with either a scalpel or a luxator. Luxators of varying sizes were used to stretch the Sharpey’s fibers and loosen the tooth. The extraction process was completed using appropriate forceps. In cases of root fractures, a metal bur was used to gain or extend surgical access to the root. Fine luxators were then used to retrieve the residual root, with special care taken to remove the entire root. The alveolus was curetted to remove any granulation tissue or cysts. Bio-Oss^®^ Collagen (BO) 100 mg or 250 mg (collagenated bovine/porcine bone substitute material) was used as needed to fill the alveolus. The xenograft was applied densely but without pressure, ensuring the remaining alveolus was completely filled. The marginal gingiva was mobilized, and the wound edges were approximated and then sutured with silk 2-0 or 3-0 sutures (Seraflex, Serag-Wiessner, Naila, Germany). Postoperative antibiotics were prescribed at the surgeon’s discretion. The antibiotics prescribed were mainly amoxicillin 1000 mg (three times daily), amoxicillin and clavulanic acid 875/125 mg (three times daily), and clindamycin 600 mg (as an alternative in cases of allergies or renal impairment, three times daily), for a maximum of three days. Postoperative analgesics were recommended regularly (ibuprofen 600 mg, three times daily, adjusted for body weight). Sutures were removed after 7–10 days.

### 2.3. Data Collection, Management, and Analysis

Patient charts and radiographic exams were analyzed, and the gathered data were tabulated. Immediately after collection, the data were irreversibly anonymized. For statistical analysis, SPSS for Mac^®^ (Version 31.0, IBM, Armonk, New York, NY, USA) was used.

Length measurements in the PRs were performed in the radiological program byzz^®^ Nxt (Orangedental, Version 10.2.142, 2024). The volume of the tooth root before extraction and the alveolar volume after ridge preservation with BO prior to implantation were created using the open-source software 3D Slicer ^®^ (Brigham and Women’s Hospital, Boston, MA, USA; Version 5.3.0-2023-02-27). In cases of incomplete datasets, missing information was excluded, and the affected cases were explicitly documented and analyzed independently.

#### 2.3.1. CBCT Three-Dimensional Models for Volume Analyses

For the calculation of a model of the tooth roots before extraction, the program 3D Slicer^®^ (Brigham and Women’s Hospital, Boston, USA; Version 5.3.0-2023-02-27) was used to generate the segments “alveolus” (tooth root from apex to the crestal bone level) and “alveolar ridge” (surrounding area of the “alveolus”).

Next, the areas of each segment were marked in all three dimensions of the CBCT images. To complete the model, the “grow from seed” tool with a seed size of 2.3 was used. The initial model was then revised, and improper parts were removed using the “scissors” function. Subsequent holes were closed using the “island” function (“filling holes”). Finally, the surface was optimized with the “smoothing surface” tool using a factor of 0.46, repeated until the model appeared smooth.

**Figure 1:**
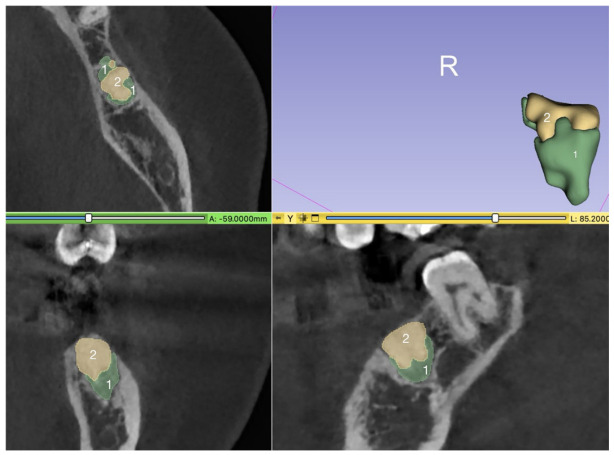
(1) “Socket” and (2) “Bio-Oss Collagen (xenograft)” within the three dimensions of the CBCT. The 3D model is presented in the upper right corner.

To calculate the models of the ridge after ridge preservation and before implantation, three segments were generated: “socket” (extraction socket), “alveolar ridge” (surrounding areas), and “Bio-Oss Collagen” (BO within the alveolus) (Figure 1).

**Figure 2:**
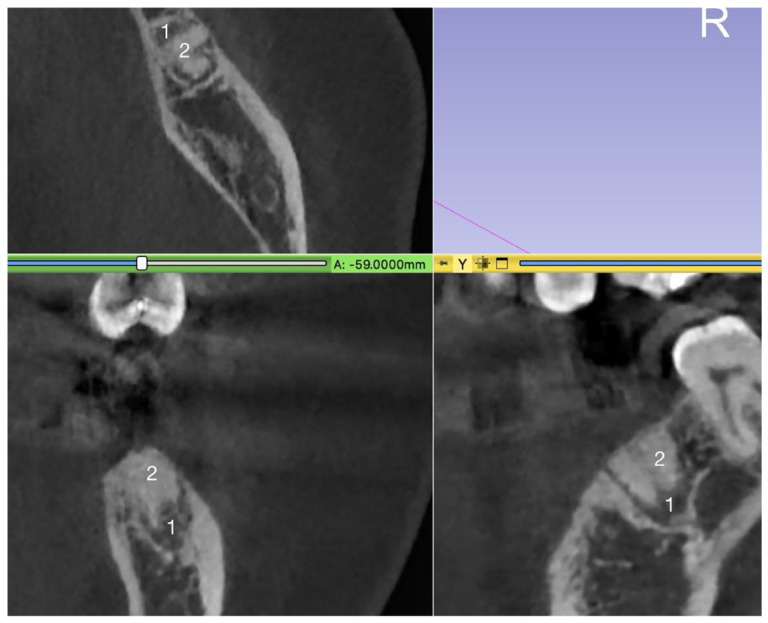
(1) Alveolus and (2) BO (xenograft), illustrating the different radiopacities.

The “xenograft (BO)” can be differentiated from the “socket” because it appears more radiopaque than both the extraction socket and normal bone structure in the CBCT. Additionally, the “socket” appears less radiopaque than the mineralized bone (Figure 2).

#### 2.3.2. Volume Calculation

The volume of the segments (“alveolus” or “socket” and “xenograft”) was calculated using 3D Slicer^®^ (Brigham and Women’s Hospital; Boston; USA; Version 5.3.0-2023-02-27). The total volume of the alveolus after ridge preservation was calculated by adding the volumes of the segments “socket” and “xenograft (BO)”.

If no CBCT was available prior to tooth extraction, the estimated tooth volume was computed based on the PR before extraction [37]. A previously validated estimation protocol was used [37]. Because the validated correlation factor applies only to molars, it could not be used to estimate premolar volumes in this study without CBCT data [37]. To extend the method to premolars, the same principle as for molars was applied, and an appropriate, dimensionless correlation factor (Appendix A) [37] was calculated. This factor (5.38) was derived from the eight premolars for which both pre-extraction CBCT and panoramic radiographs were available, allowing us to extrapolate the molar-based model to premolar sites (Appendix A) [37].

The detailed methodology, measurement procedure, and pre-extraction premolar volume results derived from panoramic radiographs are provided in Appendix A.

### 2.4. Statistics

The statistical analysis was performed by SPSS for Mac^®^ (Version 31.0, IBM, USA). Descriptive and inferential statistics were computed.

Differences between the volumes were determined using parametric statistics (*t*-test, ANOVA). Missing preoperative volumes were handled by case-wise exclusion for analyses requiring paired measurements. The significance was set at *p* < 0.05. Normality of continuous variables was assessed using the Kolmogorov–Smirnov and Shapiro–Wilk tests and supported by visual inspection of histograms and Q–Q plots. The variable “age” showed a minor deviation from perfect normality; the distributions for the volumetric measurements were approximately normal and met the assumptions for parametric testing.

A post hoc power analysis was conducted to assess whether the observed effect size and the available sample provided sufficient statistical power for the paired comparison (G-Power 2) [40]. The Pearson correlation coefficient was calculated to assess the correlation between the volumes. A regression analysis was conducted to evaluate and control for potential effects of covariates on the outcome variable.

## 3. Results

The sample was composed of 80 subjects, and 43 were male, with an average age of 59 ± 12.55 years (Table 1). The descriptive analyses were performed on the full cohort (n = 80), whereas all comparative pre-/postoperative volumetric analyses were conducted exclusively in a subset with complete imaging data (n = 60).

In 20 subjects, premolars were extracted, of which 10 were located in the maxilla and 10 in the mandible (Table 1). Sixty-one teeth were molars, with 11 located in the maxilla and 49 in the mandible (Table 1). The most common reason for tooth extraction was failed endodontic treatment with apical periodontitis or radicular cyst formation. Antibiotic prophylaxis was prescribed for 69 subjects. The vast majority of the sample were nonsmokers. Relevant medical conditions (diabetes, renal failure, immunosuppression) were recorded in 10 subjects, and 19 subjects received anticoagulant and/or antithrombotic therapy.

In 17 cases, a CBCT scan was performed prior to extraction. A digital PR existed in 43 cases. In 20 subjects, only an analog PR or a periapical radiograph was available prior to surgery, which could not be evaluated in the prescribed manner. Therefore, these subjects had to be excluded from the comparative analyses, and a subset of 60 subjects with complete radiological documentation was created.

As stated, the comparative volume analysis was based on pre- and postoperative measurements of the included subset of 60 subjects (33 males, 27 females; mean age 59 ± 12.7 years), comprising 15 premolars and 45 molars (Table 2). However, postoperative augmentation volumes were available for all 80 subjects. Analyses requiring comparative measurements (e.g., defect filling and pre-/postoperative ratios) were conducted on the subset of 60 patients, whereas analyses not dependent on preoperative values (e.g., descriptive evaluation of augmentation volume) were performed using the full cohort (Table 1 and Table 2).

The average volume of the alveolus after ridge preservation (entire cohort) was 410.01 ± 192.42 mm^3^ (premolars: 203.96 ± 59.00 mm^3^; molars: 478.69 ± 171.08 mm^3^).

### Subset Analysis

The average volume of the tooth roots (predictor variable, surrogate for relevant alveolar volume) was 451.64 ± 186.86 mm^3^. This included values of premolars (192.57 ± 42.60 mm^3^) and molars (538.04 ± 125.31 mm^3^, Table 3).

The average volume after ridge preservation was 394.86 ± 174.85 mm^3^ (premolars: 187.92 ± 57.22 mm^3^; molars: 463.84 ± 143.15 mm^3^, Table 4). As mentioned in the Materials and Methods Section, the measurement included the periapical region and the periodontal ligament, which were not part of the root volume that served as the predictor variable. Of this volume, on average, 334.70 ± 175.36 mm^3^ was augmented with BO within the alveolus.

These volumes were only slightly smaller or equal to the preoperative root volumes (Table 3).

The difference between the root volume and the alveolar volume after ridge preservation ranged from 3% to 18%, depending on tooth type (as expected) and the patients’ underlying medical conditions (*p* < 0.008). These variables significantly affected the alveolar volume after ridge preservation (Table 4). All other variables were not significantly associated with the postoperative alveolar volume (Table 4).

A significant decrease between the predictor and outcome variable volumes was noted (*p* < 0.001, *t*-test) (Table 5). The main volumetric decrease occurred in maxillary molar sites.

**Figure 3:**
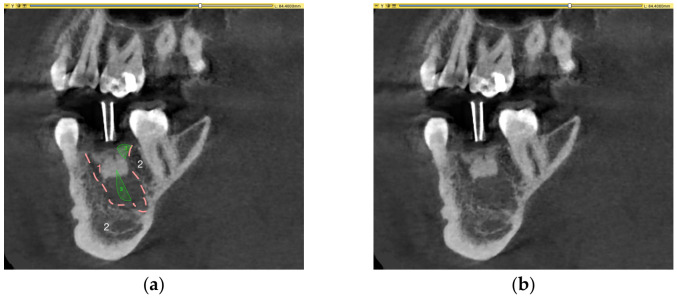
(**a**) CBCT with marked alveolus augmented with BO (1), surrounding jawbone (2) and defects that are part of the alveolus, contributing to an apparent volume gain (3); (**b**) part of the CBCT without marks.

In 13 cases, a relative volume gain was observed. The volume increase was due to alveolar volume changes during extraction (e.g., loss of interdental septum, Figure 3), excessive filling of the periodontal ligament space, and/or periradicular osteolytic lesions that were not considered in the preoperative root volume calculation (Appendix B). Additional illustrations of possible volume gain are provided in Appendix B.

In the multivariable regression model, none of the covariates reached statistical significance (all *p* > 0.05, Table 6). Anticoagulant therapy showed the largest coefficient (B = 110.1, *p* = 0.075) but did not meet the threshold for statistical significance. Antibiotic administration, sex, smoking status, and cause of tooth loss demonstrated no meaningful associations with postoperative alveolar volume.

A post hoc power analysis was performed for the paired comparison between preoperative root volume and postoperative alveolar volume after ridge preservation. Using the observed effect size (Cohen’s d = 0.63), a minimum sample size of 22 matched pairs would have been sufficient to achieve a statistical power of at least 80% at α = 0.05. As the present analysis included 60 matched pairs, the study was adequately powered for the primary volumetric comparison.

The bone volume obtained through the ridge preservation procedure was sufficient for implant placement in 78 cases. In the remaining two cases, the bony quality of the augmented areas was too low to achieve primary implant stability.

## 4. Discussion

This study aimed to measure the achievable alveolar volume of ridge preservation procedures using a bovine/porcine xenogenic bone graft. The null hypothesis was that the pre-extraction alveolar volume (surrogate root volume) can be maintained by ridge preservation procedures.

The study sample included 80 subjects (both male and female), of whom 20 premolars and 60 molars qualified for extraction and had complete data for postoperative alveolar volume measurement. A subset of 60 subjects provided sufficient radiological data for pre-extraction alveolar volume analysis. This subset comprised 45 molars and 15 premolars, which were evaluated to compare pre-extraction defect volume with postoperative augmentation volume.

The study showed that significant alveolar resorption of 3–18% occurred despite ridge augmentation. The most extensive volume reduction was noted for maxillary molar sites (18%). Therefore, the null hypothesis was rejected. The resorption kinetics were dependent on the tooth type (premolar/molar) and the tooth location (maxillary premolar/maxillary molar/mandibular premolar/mandibular molar), while the univariate statistics indicated a dependency on the general medical condition. However, the regression analysis did not reveal any independent influence of the covariates on the alveolar volume after ridge preservation. This may indicate that the initial significance was likely driven by confounding factors such as tooth type or defect size. Although subgroup sizes were small for some variables, the overall sample size was adequate for the regression model, as confirmed by the post hoc power analysis. The absence of significance in the adjusted model, therefore, reflects the elimination of confounding factors rather than a lack of statistical power.

The efficacy of ridge preservation procedures in preventing alveolar bone resorption has been described previously [18,19,20]. A wide range of materials has been proposed and studied for these procedures, including the bovine/porcine material BO [26]. Histologic evidence indicates that ridge preservation materials are either resorbed or integrated into the newly formed bone to varying degrees [24,33]. Despite all augmentation efforts, a residual amount of bone resorption after tooth extraction must be expected [4,5,10]. These findings are supported by this study’s results, which show that alveolar resorption of 3–18% can occur, depending on various influencing factors and sites. These rates seem acceptable compared to resorption rates in alveolar structures without ridge preservation, with bucco-oral loss around 40 to 60% and vertical loss of approximately 10 to 20% [10,11,14].

From a clinical perspective, the exact alveolar volumes after ridge preservation are not relevant. It is important to determine whether an implant can be placed in the augmented site without additional augmentation surgery, as this would be burdensome for the patient. In this study, the bone generated by ridge preservation was sufficient for implantation in 78/80 subjects.

The following limitations of this study should be acknowledged. Firstly, the study was retrospective, and the distribution of the included tooth types (molar/premolar and maxilla/mandible) was uneven, which may have introduced bias into the dataset. However, the sample was from a clinical setting with broad inclusion criteria, thereby enhancing the clinical relevance of the results. Only cases with clinically available radiographic datasets could be included in the paired volumetric analyses, which might have exacerbated the selection bias. Subjects with incomplete preoperative imaging were excluded only from analyses requiring matched measurements but remained part of the full-cohort descriptive evaluation. This approach ensured methodological consistency while acknowledging that imaging-based inclusion may affect internal validity.

The pre-extraction root volume, which was used as a surrogate for the ideal alveolar volume, did not include the PDL and/or periapical/periodontal osteolytic lesions. This might have led to an underestimation of the true pre-extraction defect size and therefore to an overestimation of the ridge preservation success.

In some cases, a higher alveolar volume after ridge preservation was observed due to bone fill in previously lytic or void areas (periradicular lesions and periodontal ligament). These findings might have slightly skewed the results. The measured volume gains are most likely measurement-related artifacts rather than true biological volume gains. Those cases were identified and analyzed individually. They represent a small part of the cohort.

The sample size was 80 subjects (subset for comparative analyses: 60 subjects), which is comparable to those in similar studies [24,34,41]. A post hoc power analysis was performed and confirmed sufficient power, even when only the subset was considered. Literature searches revealed no other study examining the alveolar volume after ridge preservation in humans with BO with a comparable sample size.

The methodology of this study, including various radiographic techniques for volume analysis, may be debatable. However, the described analytic techniques are based on sound and validated scientific measures [37,39], and the use of panoramic radiographs represents an ethical radiological approach, as it avoids unnecessary radiation while still providing limited volumetric information. The data analysis may depend on the examiner. Therefore, inherent inaccuracies cannot be completely ruled out. The calibration factor for premolar volume estimation was derived from a relatively small subset; however, it represents a direct extension of a previously validated geometric model for molars [37]. Although the factor shows some variability (5.38 ± 1.94), its influence on the volumetric estimates is minimal, as reflected by the small difference between CBCT-measured and PR-estimated premolar volumes (7.85 mm^3^ on average; average premolar root volume, 192.57 ± 42.60 mm^3^). A subgroup comparison between CBCT-based and PR-estimated cases was not part of this study. It is unlikely that these inaccuracies would have impacted the clinical significance of the results.

## 5. Conclusions

Within the limitations of this study, the following conclusions can be drawn. Ridge preservation procedures with a collagenated bovine/porcine bone xenograft were associated with a reduced post-extraction alveolar resorption in this study. The resorption rate was 3–18%. Most cases in the study population proceeded to implant placement without further augmentation; however, this finding is specific to our cohort and should not be interpreted as evidence that ridge preservation is universally sufficient for implant placement. Further (prospective) studies are needed to validate these observations.

## Figures and Tables

**Table 1:** Study variables with variable type and descriptive statistics of the complete study cohort.

Variable	Descriptive Statistics
Sample size	80
Sex	
Male	43 (53.7%)
Female	37 (46.3%)
Age	59 ± 12.55 years
Tooth type	
Premolar	20 (25.0%)
Molar	60 (75.0%)
Tooth location	
Maxilla premolar	10 (12.5%)
Maxilla molar	11 (13.7%)
Mandible premolar	10 (12.5%)
Mandible molar	49 (61.3%)
Cause of tooth loss	
Caries	11 (13.7%)
Periodontitis	12 (15.0%)
Fracture	12 (15.0%)
Apical Periodontitis	31 (38.8%)
No endodontic treatment possible	2 (2.5%)
Multiple	12 (15%)
Antibiotic use	
Yes	73 (91.3%)
No	7 (8.7%)
Smoker	
Yes	13 (16.3%)
No	67 (83.7%)
Anticoagulant	
Yes	17 (21.3%)
No	63 (78.7%)
Medical condition	
Yes	10 (12.5%)
No	70 (87.5%)

**Table 2:** Variables and descriptive statistics for the subset for comparative analyses (n = 60).

Variable	Subset (n = 60)
Sex	
Male	33 (55%)
Female	27 (45%)
Tooth type	
Premolar	15 (25%)
Molar	45 (75%)
Tooth placement	
Maxilla premolar	8 (13.3%)
Maxilla molar	7 (11.7%)
Mandible premolar	7 (11.7%)
Mandible molar	38 (63.3%)
Cause of tooth loss	
Caries	6 (10%)
Periodontitis	10 (16.7%)
Fracture	10 (16.7%)
Apical Periodontitis	28 (46.7%)
No endodontic treatment possible	2 (3.3%)
Multiple	4 (6.6%)
Antibiotic use	
Yes	56 (93.3%)
No	4 (6.7%)
Smoker	
Yes	11 (18.3%)
No	49 (81.7%)
Anticoagulant	
Yes	13 (21.7%)
No	47 (78.3%)
Medical condition	
Yes	6 (10%)
No	54 (90%)
Age (years)	59 ± 12.70
Sample size	60/80 (75%)

**Table 3:** Volume before extraction in mm^3^ and independent *t*-test (subset analysis).

Variable	Volume Before Extraction (mm^3^)	Independent *t*-Test *p*-Value
**Sample size (n = 60)**	451.64 ± 186.86	Not applicable
**Sex**		0.867
Male	546.35 ± 93.66	
Female	531.40 ± 147.44	
**Tooth type**		
Premolar	192.57 ± 42.60	
Molar	538.04 ± 125.31	<0.001
**Tooth location**		<0.001 ANOVA
Maxilla premolar	195.20 ± 33.40	
Maxilla molar	470.41 ± 99.92	
Mandible premolar	220.42 ±102.03	
Mandible molar	544.76 ± 137.32	
**Cause of tooth loss**		0.524 ANOVA
Caries	377.08 ± 274.21	
Periodontitis	461.02 ± 195.06	
Fracture	548.54 ± 150.35	
Apical Periodontitis	429.99 ± 171.75	
No endodontic treatment possible	397.86 ± 281.52	
Multiple	476.17 ± 194.26	
**Antibiotic use**		0.826
Yes	453.07 ± 188.93	
No	431.57 ± 178.11	
**Smoker**		0.203
Yes	526.84 ± 128.81	
No	437.00 ± 195.63	
**Anticoagulant**		0.027
Yes	351.16 ± 143.68	
No	479.43 ± 189.10	
**Medical condition**		<0.001
Yes	260.22 ± 171.26	
No	472.90 ± 177.47	

**Table 4:** Volume after ridge preservation in mm^3^ and independent *t*-test (subset analysis).

Variable	Volume After Ridge Preservation (n = 60)	Independent t-Test (*p*-value)
**Sample size**		
(n = 60)	394.86 ± 174.85	Not applicable
**Sex**		0.360
Male	413.72 ± 195.34	
Female	371.81 ± 146.33	
**Tooth type**		<0.001
Premolar	187.92 ± 57.22	
Molar	463.84 ± 143.15	
**Tooth position**		<0.001 ANOVA
Maxilla premolar	192.07 ± 63.50	
Maxilla molar	381.96 ± 81.38	
Mandible premolar	199.86 ± 73.70	
Mandible molar	475.85 ± 152.26	
**Cause of tooth loss**		0.628 ANOVA
Caries	343.92 ± 303.92	
Periodontitis	414.95 ± 141.43	
Fracture	475.79 ± 160.16	
Apical periodontitis	381.32 ± 158.52	
No endodontic treatment possible	375.14 ± 249.72	
Multiple	323.38 ± 164.38	
**Antibiotic use**		0.521
Yes	398.77 ± 177.75	
No	340.09 ± 133.14	
**Smoker**		0.370
Yes	438.01 ± 187.54	
No	385.17 ± 172.42	
**Anticoagulant**		0.099
Yes	324.00 ± 121.22	
No	414.46 ± 183.22	
**Medical condition**		0.008
Yes	217.07 ± 87.54	
No	414.54 ± 171.36	

**Table 5:** Volume before extraction vs. volume after ridge preservation, *t*-test (subset analysis).

	Volume Before Extraction	
Volume after ridge preservation	<0.001	*t*-test (*p*-value)

**Table 6:** Regression analysis of the volume after ridge preservation (subset analysis).

Predictor Variable	B (Unstandardized Coefficient)	Standard Error	Beta (Standardized Coefficient)	t-Value	Significance (*p*)
Constant	207.476	259.877	—	0.798	0.428
Antibiotic (yes/no)	–35.929	96.373	–0.052	–0.373	0.711
Sex (male/female)	69.250	51.348	0.199	1.349	0.183
Periodontitis	–1.686	15.156	–0.016	–0.111	0.912
Smoker (yes/no)	–39.109	66.240	–0.087	–0.590	0.557
Anticoagulant (yes/no)	110.095	60.588	0.262	1.817	0.075

**Figure A1:**
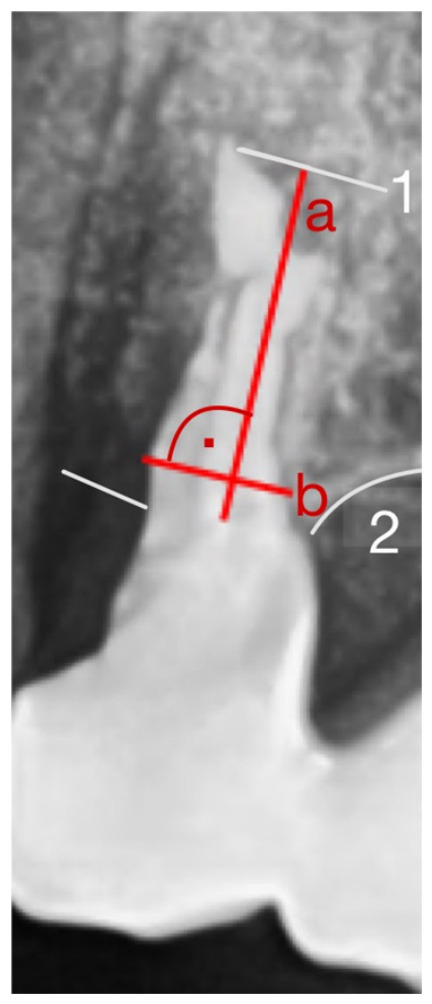
Length (a) and width (b) measurements within the PR for premolars: (1) apex and (2) crestal bone level.

**Figure A2:**
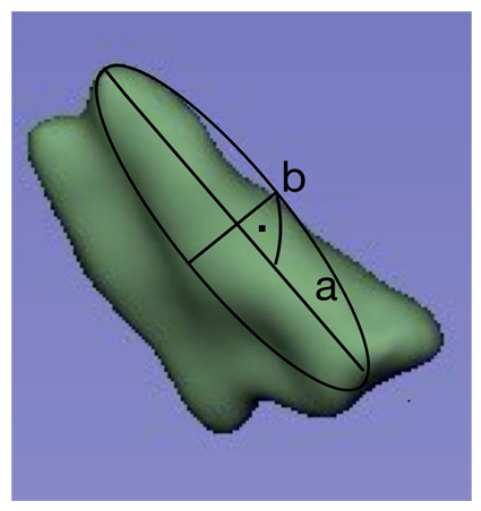
3D tooth model calculated from CBCT before extraction and elliptical surface with tooth length (a) and width (b).

**Figure A3:**
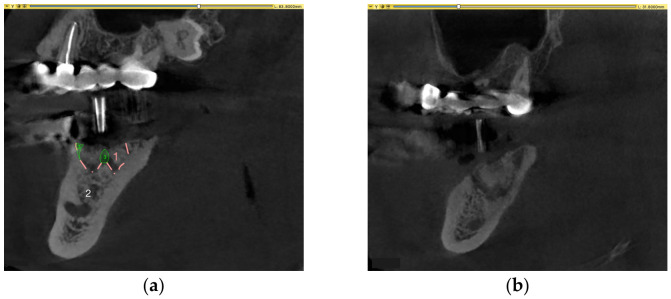
(**a**) CBCT with marked alveolus augmented with BO (1), surrounding jawbone (2) and defects that are part of the alveolus, contributing to an apparent volume gain (3); (**b**) part of the CBCT without marks.

**Figure A4:**
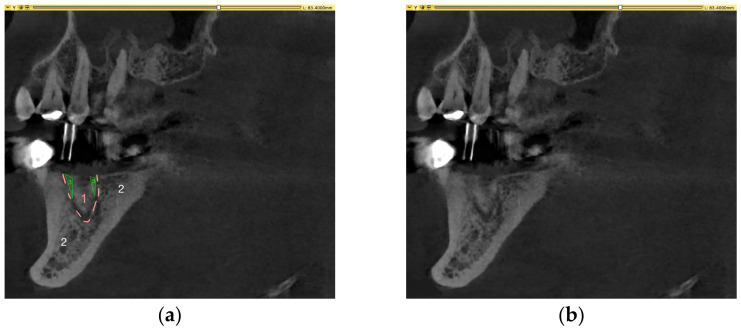
(**a**) CBCT with marked alveolus augmented with BO (1), surrounding jawbone (2) and defects that are part of the alveolus, contributing to an apparent volume gain (3); (**b**) part of the CBCT without marks.

**Figure A5:**
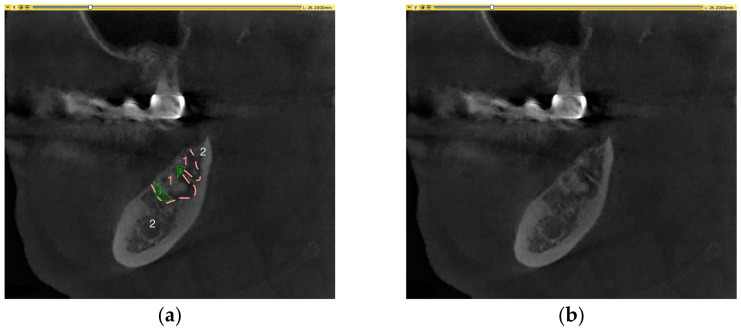
(**a**) CBCT with marked alveolus augmented with BO (1), surrounding jawbone (2) and defects that are part of the alveolus, contributing to an apparent volume gain (3); (**b**) part of the CBCT without marks.

**Figure A6:**
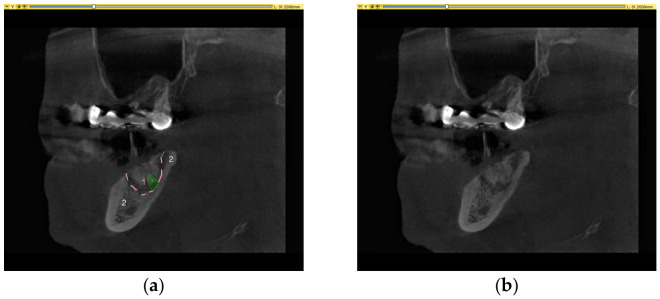
(**a**) CBCT with marked alveolus augmented with BO (1), surrounding jawbone (2) and defects that are part of the alveolus, contributing to an apparent volume gain (3); (**b**) part of the CBCT without marks.

**Figure A7:**
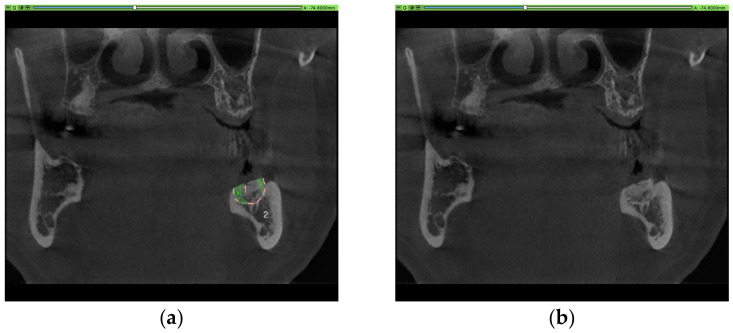
(**a**) CBCT with marked alveolus augmented with BO (1), surrounding jawbone (2) and defects that are part of the alveolus, contributing to an apparent volume gain (3); (**b**) part of the CBCT without marks.

**Figure A8:**
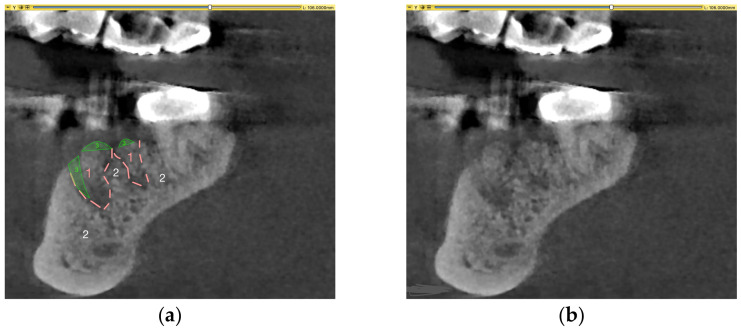
(**a**) CBCT with marked alveolus augmented with BO (1), surrounding jawbone (2) and defects that are part of the alveolus, contributing to an apparent volume gain (3); (**b**) part of the CBCT without marks.

**Figure A9:**
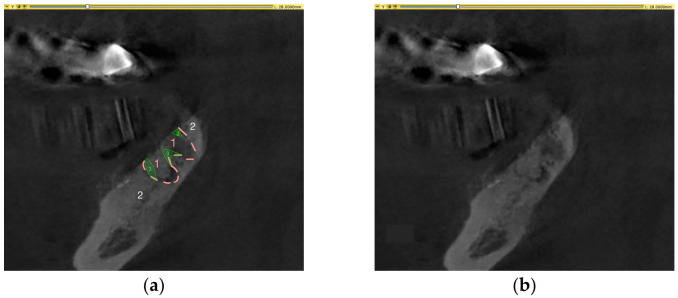
(**a**) CBCT with marked alveolus augmented with BO (1), surrounding jawbone (2) and defects that are part of the alveolus, contributing to an apparent volume gain (3); (**b**) part of the CBCT without marks.

**Figure A10:**
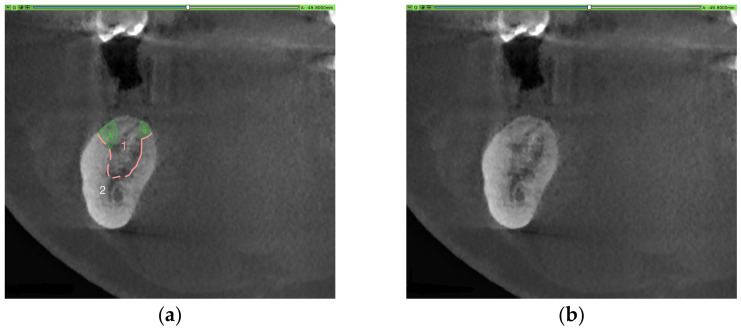
(**a**) CBCT with marked alveolus augmented with BO (1), surrounding jawbone (2) and defects that are part of the alveolus, contributing to an apparent volume gain (3); (**b**) part of the CBCT without marks.

**Figure A11:**
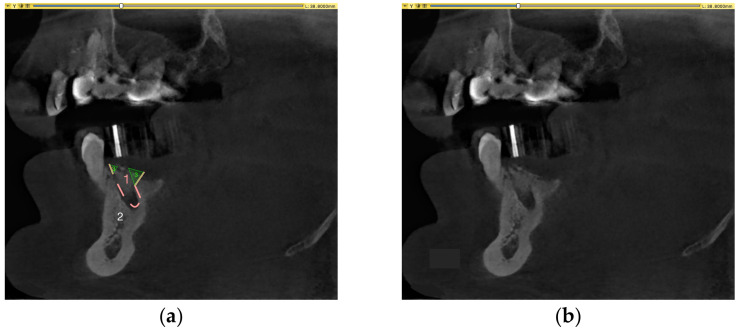
(**a**) CBCT with marked alveolus augmented with BO (1), surrounding jawbone (2) and defects that are part of the alveolus, contributing to an apparent volume gain (3); (**b**) part of the CBCT without marks.

**Figure A12:**
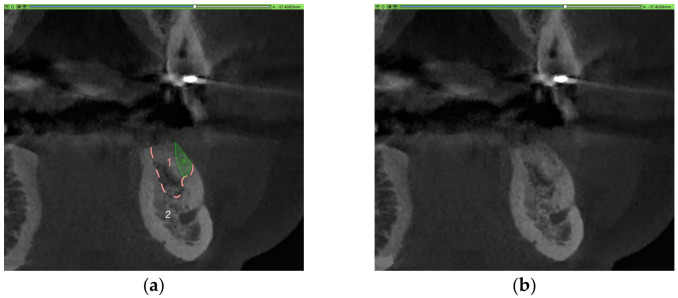
(**a**) CBCT with marked alveolus augmented with BO (1), surrounding jawbone (2) and defects that are part of the alveolus, contributing to an apparent volume gain (3); (**b**) part of the CBCT without marks.

**Figure A13:**
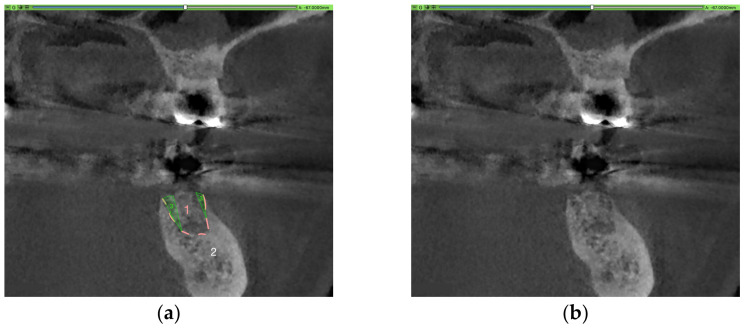
(**a**) CBCT with marked alveolus augmented with BO (1), surrounding jawbone (2) and defects that are part of the alveolus, contributing to an apparent volume gain (3); (**b**) part of the CBCT without marks.

**Figure A14:**
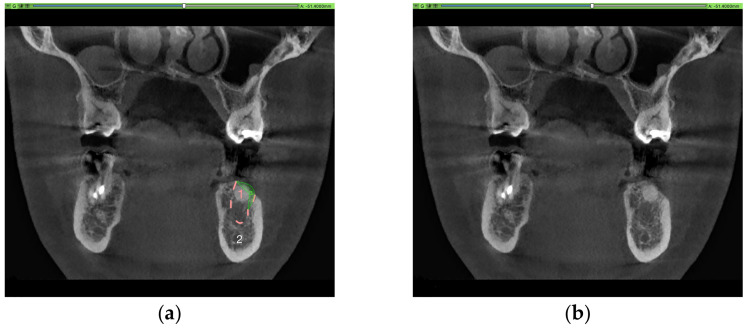
(**a**) CBCT with marked alveolus augmented with BO (1), surrounding jawbone (2) and defects that are part of the alveolus, contributing to an apparent volume gain (3); (**b**) part of the CBCT without marks.

**Figure A15:**
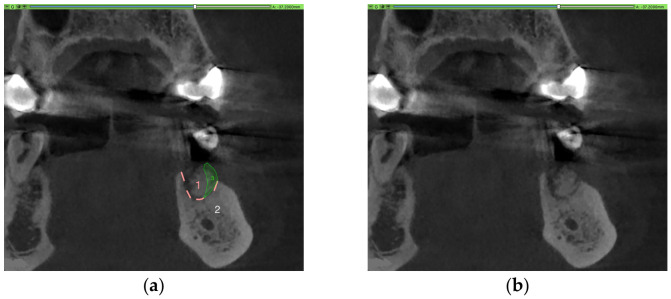
(**a**) CBCT with marked alveolus augmented with BO (1), surrounding jawbone (2) and defects that are part of the alveolus, contributing to an apparent volume gain (3); (**b**) part of the CBCT without marks.

**Figure A16:**
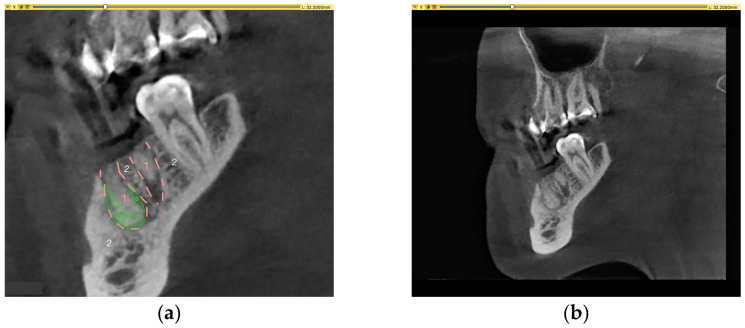
(**a**) CBCT with marked alveolus augmented with BO (1), surrounding jawbone (2) and defects that are part of the alveolus, contributing to an apparent volume gain (3); (**b**) part of the CBCT without marks.

**Figure A17:**
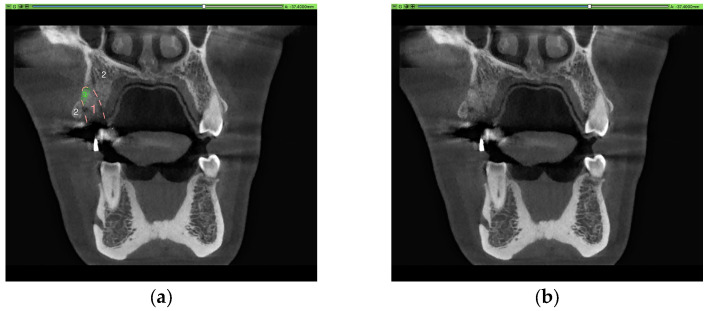
(**a**) CBCT with marked alveolus augmented with BO (1), surrounding jawbone (2) and defects that are part of the alveolus, contributing to an apparent volume gain (3); (**b**) part of the CBCT without marks.

**Figure A18:**
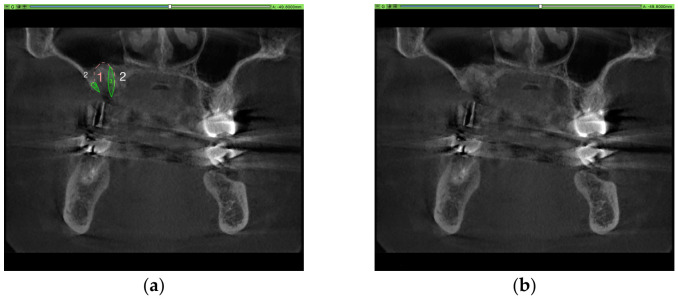
(**a**) CBCT with marked alveolus augmented with BO (1), surrounding jawbone (2) and defects that are part of the alveolus, contributing to an apparent volume gain (3); (**b**) part of the CBCT without marks.

**Figure A19:**
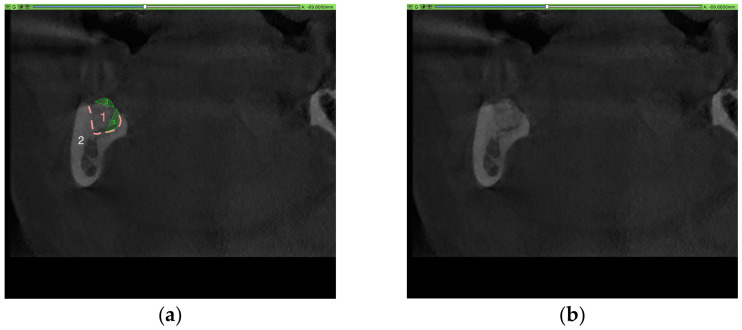
(**a**) CBCT with marked alveolus augmented with BO (1), surrounding jawbone (2) and defects that are part of the alveolus, contributing to an apparent volume gain (3); (**b**) part of the CBCT without marks.

## Data Availability

The datasets used and analyzed during the current study are available from the corresponding author on reasonable request.

## Abbreviations

The following abbreviations are used in this manuscript:

PR	Panoramic radiograph
CBCT	Cone-beam volume tomography
BO	Bio-Oss^®^ Collagen (Geistlich, Swiss)
AI	Artificial intelligence

## Appendix A

### Appendix A.1. Estimation of Premolar Root Volume from Panoramic Radiographs

For premolars without pre-extraction CBCT data, the root volume was estimated from panoramic radiographs (PRs) using an approach analogous to the previously validated molar method [37]. To establish a premolar-specific calibration factor, the subset of eight premolars for which both preoperative CBCT scans and PRs were available was used.

#### Appendix A.1.1. Measurement of Linear Dimensions on PRs

For each of the eight premolars, root length and root width were measured on the PRs from the crestal bone level to the apex (Figure A1) following the same protocol used for molars [37].

#### Appendix A.1.2. Construction of an Elliptical Reference Surface

Half of the measured length and half of the measured width were used to construct an elliptical surface area, identical to the procedure applied in the molar model (Table A1, Figure A1). This elliptical surface served as a two-dimensional surrogate for the root cross-section.

**Table A1:** Volume CBCT in mm^3^, factor (dimensionless) volume calculated with a factor of 5.38 in mm^3^, elliptical surface in mm^2^, and length and width in mm.

	Volume CBCT	Factor	Volume Calculated with Average Factor 5.38	Elliptical Surface	Length PR	Width PR
	186.46	9.16	109.47	20.35	7.78	3.33
	226.02	7.00	173.76	32.30	8.92	4.61
	262.80	4.83	292.85	54.43	15.68	4.42
	130.27	3.45	203.22	37.77	10.57	4.55
	221.90	5.50	217.04	40.34	9.84	5.22
	82.74	3.06	199.65	27.00	9.71	3.54
	190.00	5.12	199.65	37.11	9.45	5.00
	255.11	4.96	276.89	51.47	12.18	5.38
**Average:**	**194.41** **±** **61.85**	**5.38** **±** **1.94**	**202.26** **±** **61.73**	**37.59** **±** **11.47**	**10.51** **±** **2.44**	**4.50** **±** **0.74**

#### Appendix A.1.3. Calculation of True Root Volume from CBCT

For the same teeth, the true three-dimensional root volume was estimated from the preoperative CBCT scans. Using 3D Slicer^®^ (Brigham and Women’s Hospital, Boston, USA), two segments (“alveolus” and “surrounding bone”) were generated to reconstruct the root geometry and calculate its volume (Figure A2, Table A1) following the validated molar workflow [37].

#### Appendix A.1.4. Derivation of a Premolar-Specific Calibration Factor

For each premolar, the CBCT-derived root volume was divided by the corresponding elliptical surface area measured on the PR. This yielded a dimensionless calibration factor describing the relationship between the two-dimensional PR-based surrogate and the true three-dimensional root volume. Across the eight premolars, the mean factor was 5.38 ± 1.94 (Table A1).

A counter-validation was performed by recalculating the premolar volumes from the PR-based ellipses using this factor, confirming internal consistency.

#### Appendix A.1.5. Application to Premolars Without CBCT

For all premolars in the cohort lacking preoperative CBCT data, root volume was estimated by multiplying the PR-derived elliptical surface area by the calibration factor 5.38, analogous to the established molar method (Table A2) [37].

**Table A2:** Volume of the pre-extraction volume of the premolars based on PR and calculated with a factor of 5.38 in mm^3^.

Volume Premolar Based on PR
162.63
196.55
148.14
115.41
149.24
198.92
198.80
196.94
160.03

#### Appendix A.1.6. Summary

This approach extends a previously validated molar estimation method to premolars by deriving a premolar-specific calibration factor from cases with complete imaging [37]. It allows reliable approximation of premolar root volumes from panoramic radiographs when CBCT data are unavailable, ensuring methodological consistency across the dataset.

## Appendix B

### Figures Illustrating Cases of Volume Gain or No Resorption

In 13 cases within the cohort subset (out of 60 cases), the volume after ridge preservation appeared to be greater than the pre-extraction tooth root volume. These findings initially seemed unlikely. A closer examination of these cases showed that the apparent bony consolidation after ridge preservation included existing periodontal pre-extraction radiolucencies, the periodontal ligament, and periodontal bone defects, which were not accounted for in the tooth root volume measurement (used as a surrogate for alveolar volume and as a predictor variable) (Figure A3, Figure A4, Figure A5, Figure A6, Figure A7, Figure A8, Figure A9, Figure A10, Figure A11, Figure A12, Figure A13, Figure A14, Figure A15, Figure A16, Figure A17, Figure A18 and Figure A19). Examples of defects associated with extraction include the loss and subsequent bony replacement of the intra-alveolar septum (Figure A3, Figure A5, Figure A6 and Figure A9) or filling of a periodontal gap, either caused by periodontitis or extraction trauma (Figure A3, Figure A4, Figure A5, Figure A7, Figure A8, Figure A9, Figure A10 and Figure A11).

In some cases, the reconstruction of the buccal alveolar wall was achieved by ridge preservation with BO (Figure A10, Figure A12, Figure A13, Figure A14 and Figure A15).

Periapical and periodontal osteolytic lesions, which were explicitly excluded from the original tooth root volume measurement by definition, could not be clearly differentiated from the original defect after ridge augmentation (Figure A16 and Figure A17).

In rare cases, an actual volume gain can be observed when alveolar envelopes exceed the crestal area (Figure A7, Figure A8, Figure A18 and Figure A19).

It can be stated that a larger volume after extraction is not automatically a volume gain; in most cases, it results from a defect larger than the original root volume, and as such, it is reasonable. The cases in which BO augmentation enabled bone growth outside the bony envelope warrant further investigation and could be a sign of an osteoconductive character of the material.
